# Up-regulation of lncRNA SNHG1 indicates poor prognosis and promotes cell proliferation and metastasis of colorectal cancer by activation of the Wnt/β-catenin signaling pathway

**DOI:** 10.18632/oncotarget.22903

**Published:** 2017-12-04

**Authors:** Yuping Zhu, Bo Li, Zhuo Liu, Lai Jiang, Gang Wang, Min Lv, Dechuan Li

**Affiliations:** ^1^ Department of Colorectal Surgery, Zhejiang Cancer Hospital, Hangzhou 310022, Zhejiang Province, China; ^2^ Department of Ultrasonic, Zhejiang Cancer Hospital, Hangzhou 310022, Zhejiang Province, China

**Keywords:** colorectal cancer, SNHG1, Wnt/β-catenin, proliferation, metastasis

## Abstract

Recently, the lncRNA small nucleolar RNA host gene (SNHG1) has been exhibited to be upregulated, which plays a crucial role in the development and prognosis of several cancers. However, the role of the biology and clinical significance of SNHG1 in the tumorigenesis of colorectal cancer (CRC) has rarely been reported. In this work, we firstly found that SNHG1 expression levels were upregulated aberrantly in colorectal cancer tissues and colorectal cancer cell lines. By Kaplan-Meier survival analysis, patients with high SNHG1 expression level had poorer overall survival (OS) and progression-free survival (PFS) than those with low SNHG1 expression. In multivariate analysis, increased SNHG1 expression was proved to be an independent unfavorable prognostic indicator for CRC. *In vitro* experiments revealed that SNHG1 silencing inhibited the growth and metastasis and induced apoptosis of CRC cell lines. Finally, we found that SNHG1 may induce the activation of the WNT/β-catenin pathway through regulating β-catenin expression and transcription factor-4 (TCF-4), cyclin D1 and MMP-9. Altogether, our findings demonstrated that lncRNA SNHG1, was high expressed in colorectal cancer tissues and may serve as a tumor oncogene through regulating WNT/β-catenin signal pathway, which provided a candidate diagnostic biomarker and a promising therapeutic target for patients with CRC.

## INTRODUCTION

Colorectal cancer (CRC) is a common cause of cancer-related mortality worldwide [1]. Because patients with CRC are very often diagnosed at advanced stages and the progression of the disease is rapid, the 5-year relative survival for colorectal cancer is still poor [2, 3]. With the numerous molecular and pathological studies, colorectal cancer is now regarded as a heterogeneous group of diseases with definite clinical-pathological features, which arises from the dysregulation of many cancer-associated genes [4]. Thus, a better insights of the molecular mechanisms on CRC initiation and progression may contribute to develop new strategy for the treatment and to meliorate the prognosis of CRC patients. Currently, increasing evidence have exhibited that noncoding RNAs (ncRNAs) may be connected with CRC pathogenesis, providing new understanding into the biology of CRC [5, 6].

LncRNAs, which are defined as ncRNAs longer than 200 nucleotides without open reading frames (ORFs) and are unable to be translated into proteins [7]. Aberrant expression of lncRNAs have been demonstrated in a variety of pathological processes in many species [8, 9]. Furthermore, emerging evidence suggests that lncRNAs serve as critical regulators of cancer initiation, progression, and metastasis [10, 11]. The small nucleolar RNA host gene (SNHG1, GenBank accession ID:23642), a novel non-coding RNA localized at 11q12.3, has exhibited oncogenic role in diverse cancer. Long noncoding RNA SNHG1 predicts a poor prognosis and promotes hepatocellular carcinoma tumorigenesis [12] and SNHG1 exacerbates hepatocellular carcinoma through suppressing miR-195 [13]. Wang et al. has reported that upregulation of the long non-coding RNA SNHG1 predicts poor prognosis, promotes cell proliferation and invasion, and reduces apoptosis in glioma [14]. Moreover, lncRNA SNHG1 negatively regulates miR-199a-3p to enhance CDK7 expression and promote cell proliferation in prostate cancer [15] and can serve as diagnostic and prognostic markers for prostate cancer through androgen-responsive manner [16]. Meanwhile, lncRNA SNHG1 is upregulated and contributes to progression of non-small cell lung cancer through inhibition of miR-101-3p and activation of Wnt/β-catenin signaling pathway [17, 18].

However, it is still unknown whether this distinct function of lncRNA SNHG1 is involved in the tumorigenesis of CRC. In our present study, we explored the expression, clinical significance, biological function and molecular mechanism of lncRNA SNHG1 in CRC. For the first time, our results demonstrated a novel lncRNA signaling pathway regulatory network that is the SNHG1-β-catenin-WNT signaling pathway in CRC.

## RESULTS

### Expression of SNHG1 was increased in colorectal cancer and cells and associated with the clinicopathological factors in patients with colorectal cancer

**Figure 1:**
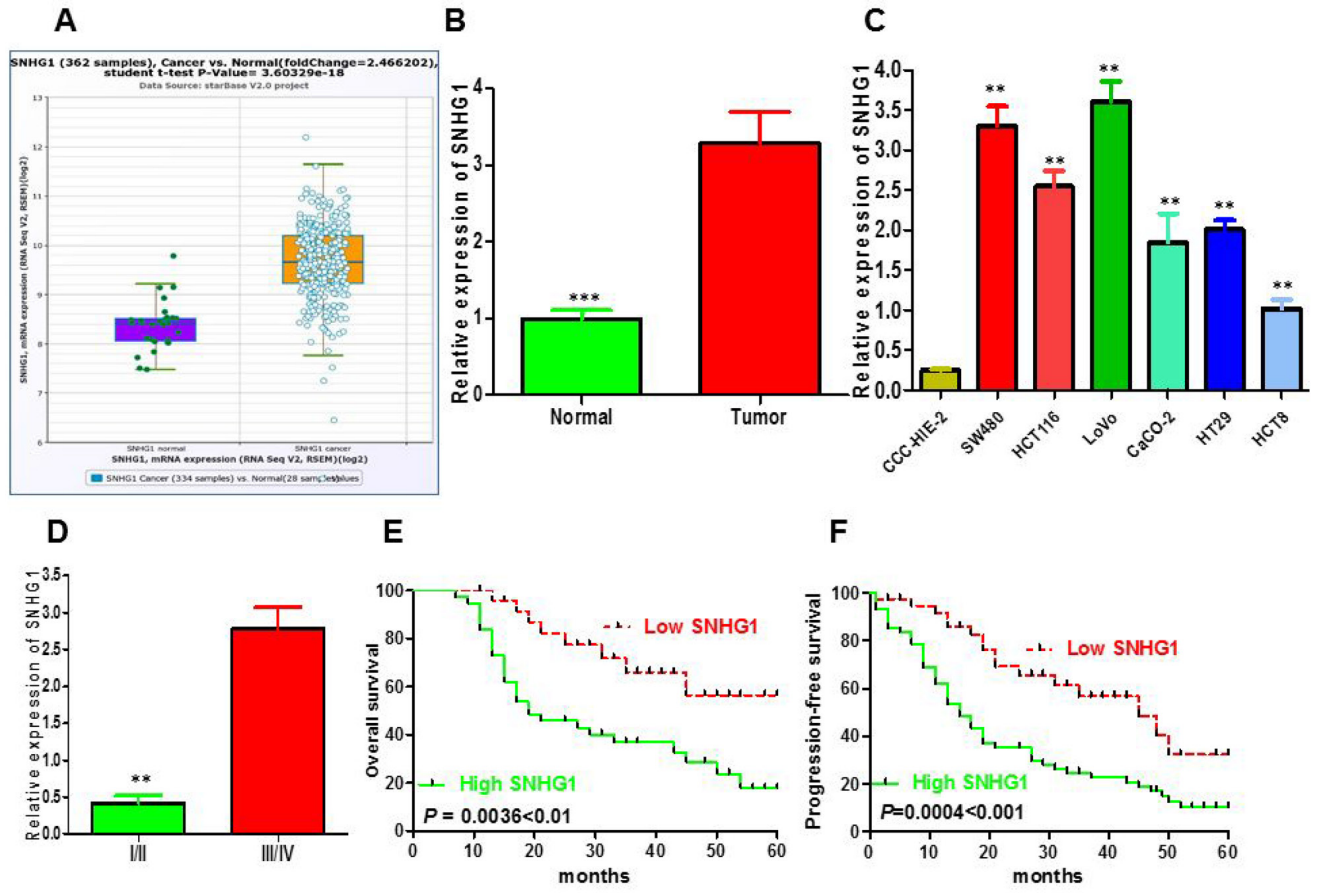
LncRNA SNHG1 expression was up-regulated and its clinical parameters in colorectal cancer **(A)** The expression of SNHG1 in normal or CRC from The Cancer Genome Atlas (TCGA) Data Portal from starBASE v2.0. **(B)** Relative expression of SNHG1 in 108 pairs of colorectal cancer tissues and adjacent non-tumor tissues by qRT-PCR analysis. ^***^P<0.001 compared with non-tumor control. **(C)** The expression levels of SNHG1 in a panel of colorectal cancer cell lines were determined by qRT-PCR and compared with that in normal intestinal mucous cell line(CCC-HIE-2). ^**^P < 0.01 compared with the CCC-HIE-2 cell. **(D)** Higher SNHG1 was positively correlated with TNM stage. **(E)** Patients with high levels of SNHG1 expression showed reduced survival times compared with patients with low levels of SNHG1 by Kaplan-Meier overall survival curves (*P*=0.0036<0.01). **(F)** Kaplan-Meier progression-free survival curves for two groups defined by low and high expression of SNHG1 in patients with colorectal cancer (*P*=0.0004<0.001). Data represent the mean ± SD from three independent experiments. ^*^P < 0.05; ^**^P < 0.01.

To detect whether lncRNA SNHG1 was involved in the progression of CRC, we determined SNHG1 expression in TCGA Data Portal from starBASE v2.0. The result showed that SNHG1 was higher expression in tumor tissues compared to normal tissues in CRC (Figure 1A; *P* < 0.05). We then confirmed that the relative expression level of SNHG1 in colorectal cancer tissues (n=104) compared to corresponding normal counterparts (n=104) by qRT-PCR, and normalized to GAPDH. As shown in Figure 1B, the SNHG1 level was potently augmented in colorectal cancer tissues compared to corresponding normal counterparts (*P*<0.001, Figure 1B). Moreover, up-regulated SNHG1 expression was strikingly correlated with advanced TNM stage (Figure 1D, *P*<0.01). Then, we explored the expression of SNHG1 in a panel of colorectal cancer cells (SW480, HCT116, HT-29, LOVO and CaCO-2) and normal intestinal mucous cell line (CCC-HIE-2) by qRT-PCR. As shown in Figure 1C, the result of qRT-PCR indicated that the colorectal cancer cells exhibited high expression of SNHG1 compared to the normal intestinal mucous cell line (*P*<0.01, Figure 1C). These data clarified that upregulated SNHG1 might be involved in colorectal cancer carcinogenesis. According to the median value of relative SNHG1 expression in cancer tissues, colorectal cancer patients were classified into two groups: relative high-SNHG1 expression group (n=54) and relative low-SNHG1 expression group (n=54). This classification was based on published study [19, 20]. The relationship of SNHG1 expression level with the clinicopathological factors in colorectal cancer was assessed (Table 1). High SNHG1 expression was more frequently to be detected in tumors with bigger tumor diameter (*P*=0.012), advanced TNM stage (*P*=0.004), lymph node metastasis (*P*=0.011), deep invasion (*P*=0.009). While no statistically significant correlations were observed between SNHG1 expression and age (*P*=0.439), gender (*P*=0.326), histological differentiation (*P*=0.491), distant metastasis (*P*=0.339), primary tumor site (*P*=0.839), obstruction (*P*=0.279).

**Table 1:** The association between SNHG1 expression and clinicopathological parameters in colorectal cancer

Characteristics	Number	Expression of SNHG1		*P* value
	n	Low expression	High expression	
Age(years)				
<50	48	26	22	0.439
≥50	60	28	32	
Gender				
Male	65	35	30	0.326
Female	43	19	24	
Tumor diameter (cm)				
<4	38	25	13	0.012^*^
≥4	70	29	41	
Tumor differentiation				
Well-moderate	62	27	35	0.491
Poor	46	17	29	
TNM stage				
I-II	47	31	16	0.004^**^
III-IV	61	23	38	
Lymph node metastasis				
Positive	65	26	39	0.011^*^
Negative	43	28	15	
Venous invasion				
Present	20	9	11	0.620
Absent	88	45	43	
Nervous invasion				
Present	18	8	10	0.606
Absent	90	46	44	
Depth of invasion				
T1 - T2	39	26	13	0.009^**^
T3 - T4	69	28	41	
Distant metastasis				
Present	22	9	13	0.339
Absent	86	45	41	
Primary tumor site				
Colon	71	35	36	0.839
Rectum	37	19	18	
Obstruction				
Present	92	44	48	0.279
Absent	16	10	6	

*P* value when expression levels were compared using Pearson χ2 test. TNM, tumor-node-metastasis; ^*^, *P*<0.05; ^**^ *P*<0.01.

### Upregulation of SNHG1 indicated poor prognosis in colorectal cancer patients

To examine the links between SNHG1 expression and colorectal cancer patients’ prognosis, we performed the Kaplan–Meier analysis and log-rank test to assess overall survival (OS) and progression-free survival (PFS) of patients with colorectal cancer and SNHG1 expression. As shown in Figure 1E and 1F, patients with high SNHG1 expression level had poorer overall survival (P=0.0036) and progression-free survival (P=0.0004) than those with low SNHG1 expression. To further validate the prognostic role of SNHG1 in colorectal cancer patients, we carried out the univariate and multivariate survival analysis for OS and PFS in colorectal cancer patients. As shown in Table 2, univariate analysis displayed that a few prognosis factors (distant metastasis, depth of invasion, lymph node metastasis, venous invasion, nervous invasion, obstruction, TNM stage, tumor diameter and SNHG1 expression) were statistically significant risk factors influencing OS and PFS of patients. Multivariate analysis further elucidated that SNHG1 expression could be regarded as a significant independent predictor of OS and PFS in colorectal cancer patients (Table 2). Altogether, these data displayed intensified expression of SNHG1 predominantly diminished patients’ survival time and might exhibit crucial role in the prognosis of patients with colorectal cancer.

**Table 2:** Univariate and multivariate analysis of clinic-pathologic factors for OS or PFS in colorectal cancer

Variables	OS			PFS		
	HR	95% CI	*P* value	HR	95% CI	*P* value
**Univariate analysis**						
Age (≥50 vs. <50 years)	0.943	0.876-1.223	0.686	0.952	0.884-1.107	0.720
Gender (Male vs. Female)	1.025	0.924-1.319	0.521	1.1131	0.974-1.542	0.665
Primary tumor size (Colon vs. Rectum)	1.207	0.889-1.643	0.419	1.126	0.903-1.477	0.524
Distant metastasis (P vs. N)	4.268	1.115-5.623	0.004^**^	3.408	1.184-5.823	0.007^**^
Depth of invasion (T1 + T2 vs. T3 + T4)	3.246	1.024-6.178	0.002^**^	3.652	2.136-4.894	0.002^**^
Tumor differentiation (poorly vs. well)	1.206	0.673-2.941	0.388	1.342	0.965-1.972	0.465
Lymph node metastasis (P vs. N)	4.103	2.025-5.194	0.001^**^	3.328	1.975-5.926	0.003^**^
Venous invasion(Present vs. Absent)	3.805	0.965-6.465	0.025^*^	3.201	0.972-6.320	0.016^*^
Nervous invasion(Present vs. Absent)	3.190	0.687-7.543	0.014^*^	3.424	0.763-6.879	0.012^*^
Obstruction(Present vs. Absent)	3.762	0.922-5.422	0.016^*^	3.976	0.935-6.961	0.025^*^
TNM stage (III/IV vs. I/II)	4.019	1.797-6.297	0.004^**^	5.328	1.934-6.301	0.008^**^
Tumor diameter (cm) (≥4 vs.<4)	3.612	1.262-4.374	0.006^**^	4.291	1.303-5.751	0.007^**^
SNGH1 expression (high vs. low)	5.399	2.467-6.725	0.001^**^	4.936	1.981-5.722	0.002^**^
**Multivariate analysis**						
Distant metastasis (P vs. N)	0.896	0.653-3.921	0.235	0.973	0.689-3.218	0.267
Depth of invasion (T1 + T2 vs. T3 + T4)	1.257	0.875-3.219	0.344	1.109	0.765-3.208	0.354
Tumor differentiation (poorly vs. well)	1.178	0.791-4.272	0.452	0.928	0.739-3.758	0.562
Lymph node metastasis (P vs. N)	2.732	0.945-4.032	0.015^*^	2.642	1.004-4.507	0.013^*^
Venous invasion(Present vs. Absent)	1.004	0.786-2.964	0.452	0.976	0.854-3.209	0.432
Nervous invasion(Present vs. Absent)	0.966	0.768-3.109	0.342	0.953	0.942-2.977	0.351
TNM stage (III/IV vs. I/II)	3.105	1.244-5.206	0.004^**^	3.109	1.156-4.814	0.006^**^
Tumor diameter (cm) (≥4 vs.<4)	2.113	1.105-6.082	0.003^**^	1.996	1.035-3.817	0.013^*^
SNGH1 expression (high vs. low)	3.172	1.554-6.209	0.004^**^	2.893	1.362-4.702	0.001^**^

OS, overall survival;PFS, progression-free survival; HR, hazard ratio; N, Negative; P, Positive; ^*^P< 0.05, ^**^P<0.01.

### Silencing of SNHG1 suppressed growth of SW480 and LoVo cells *in vitro*

**Figure 2:**
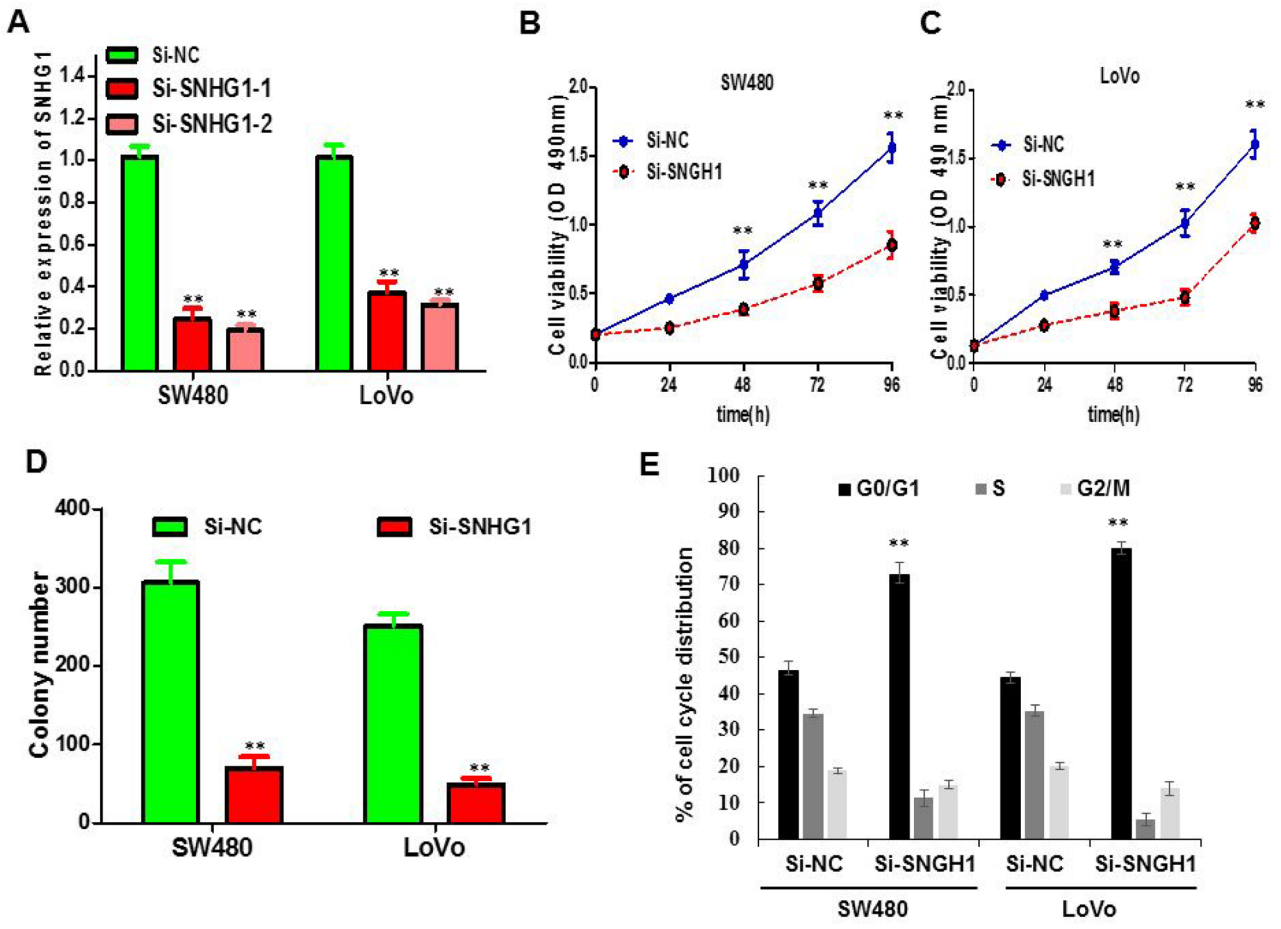
The influence of SNHG1 expression on cell viability and cell cycle **(A)** qPCR analysis of SNHG1 expression levels following the treatment of SW480 and LoVo cells with siRNAs against SNHG1. **(B)** and **(C)** CellTiter-Glo assay showed knockdown of SNHG1 inhibited cell proliferation of SW480 and LoVo cells. **(D)** Colony formation assay exhibited knockdown of SNHG1 dramatically inhibited the colony-forming ability of SW480 and LoVo cells. **(E)** Flow cytometric analysis showed that depletion of SNHG1 promoted G0/G1 phase arrest of the SW480 and LoVo cell compared with cells transfected with Si-NC. Each assay was performed in triplicate. Data are mean ± SD. ^*^ *P*<0.05, ^**^P<0.01.

We probed that lncRNA SNHG1 expression was relatively higher in SW480 and LoVo cell lines than that in HCT116, CaCO2, HT29 and HCT8 cancer cell lines (Figure 1C). Thus, we selected SW480 and LoVo cell lines for the following loss of function studies. To further investigate the role of SNHG1 in colorectal cancer cells, the lncRNA SNHG1-specific Si-SNHG1 was synthesised and transfected into SW480 and LoVo cells. As shown in Figure 2A, cells transfected with Si-SNHG1 obviously reduced mRNA expression level of SNHG1 compared with the Si-NC group in both cells by qRT-PCR (^**^P<0.01; Figure 2A). To evaluate the influence of SNHG1 on the proliferation of colorectal cancer cell *in vitro*, MTS assays verified that the silencing of SNHG1 apparently abrogated the proliferation rate of SW480 and LoVo cells (^**^P<0.01; Figure 2B and 2C). Furthermore, colony formation assay was carried out to further examine the effect of SNHG1 on growth of SW480 cells and LoVo cells. Similarly, the results indicated that colorectal cancer cells transfected with siRNA dramatically attenuated the colony numbers (^**^P<0.01; Figure 2D). In addition, flow cytometry was used to analyze cell cycle distribution of SNHG1 knockdown cancer cell. The results demonstrated that the cell population in the G0/G1 phase was boosted but the S phase population was decreased after the knockdown of SNHG1 compared to the results examined for the Si-NC cells (Figure 2E), further confirming that downregulation of SNHG1 expression might disturb cancer cell proliferation by disrupting the cell cycle. In a word, these data exhibited that SNHG1 may function as an oncogene through facilitating colorectal cancer cell growth.

### Silencing of SNHG1 promoted apoptosis of colorectal cancer cells

**Figure 3:**
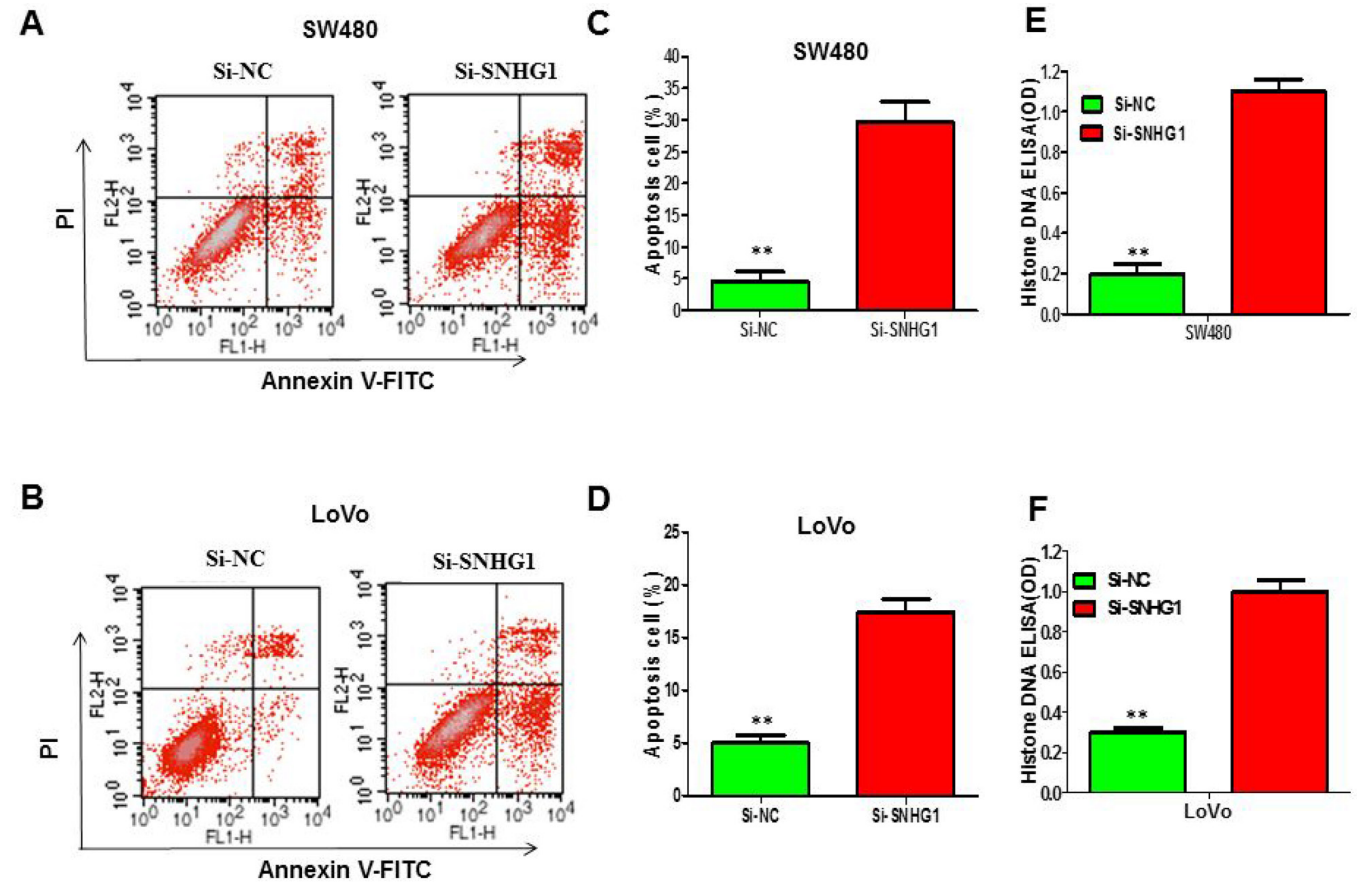
Suppression of lncRNA SNHG1 expression obviously enhanced the apoptosis of colorectal cancer cells **(A)** and **(B)** Apoptosis of SW480 and LoVo cell lines was determined by flow cytometry. **(C)** and **(D)** Histogram of percentage of apoptotic cells, according to (A) and (B). **(E)** and **(F)** Histone DNA ELISA assay was used to measure the apoptosis of SW480 and LoVo cell lines. Each assay was performed in triplicate. Data are mean ± SD. ^*^P<0.05, ^**^P<0.01.

To explore whether knockdown of SNHG1 alert cell apoptosis, we carried out flow cytometry to analyze the cell apoptosis of colorectal cancer cells when transfected with Si-SNHG1. The data displayed that knockdown of SNHG1 remarkably induced apoptosis of SW480 (Figure 3A) and LoVo cells (Figure 3B), especially early apoptosis. Compared with the cells transfected with Si-NC, cell apoptosis enlarged approximately 21.23% in SW480 cells when treated with Si-SNHG1 (Figure 3C), and 13.57% in LoVo cells (Figure 3D). Then, we performed Histone-DNA ELISA assay to further evaluate the apoptosis in colorectal cancer cells of SNHG1 knockdown. Histone-DNA ELISA assay analysis confirmed that SNHG1 knockdown exerted sharply pro-apoptosis in SW480 (Figure 3E) and LoVo cells (Figure 3F).

### Silencing of SNHG1 abrogated migration and invasion of colorectal cancer cells

**Figure 4:**
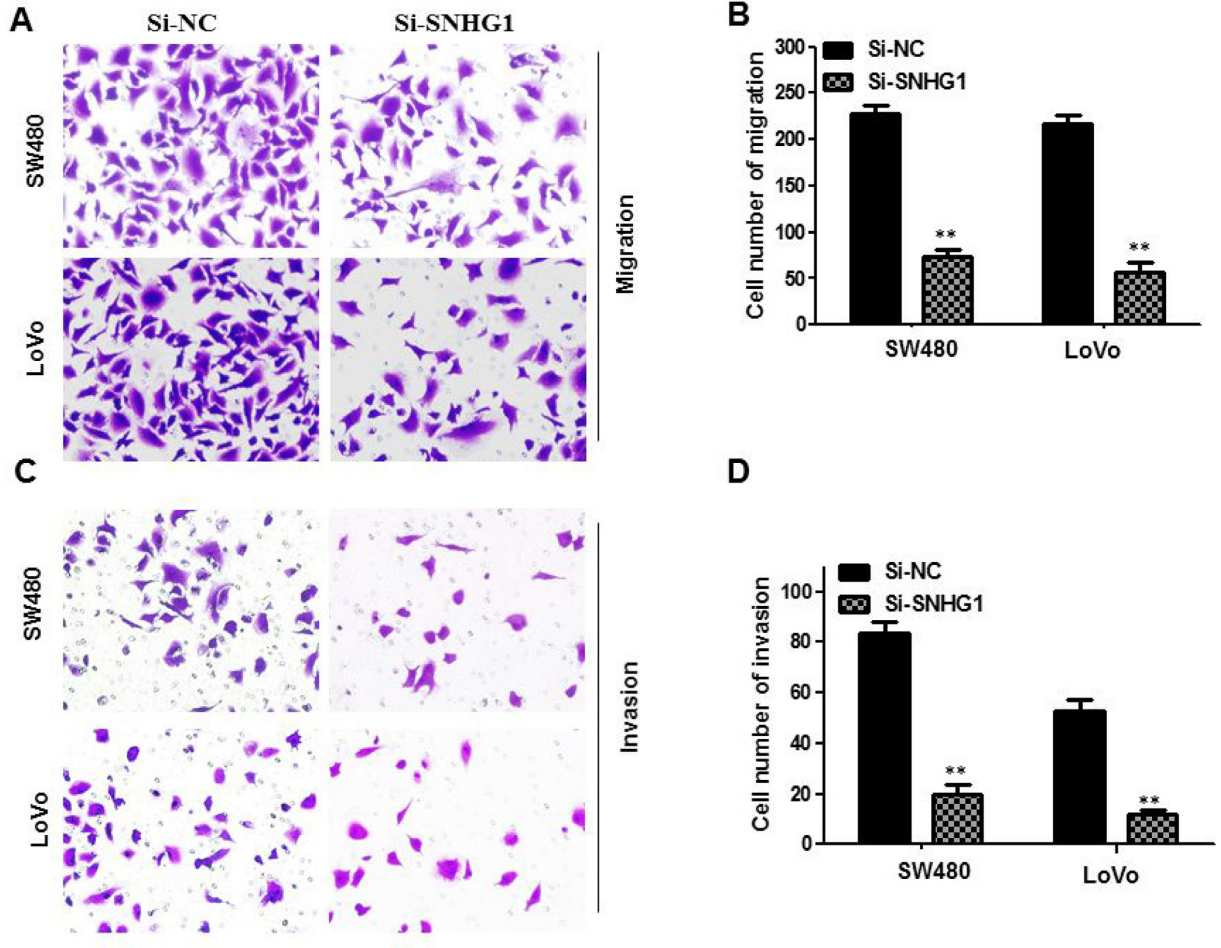
Knock-down SNHG1 attenuated migration and invasion of colorectal cancer cells Transwell assays were used to investigate the migratory and invasive abilities of si-SNHG1 transfected CRC cells. **(A)** and **(B)** Inhibition of migration of SW480 and LoVo cells by SNHG1 siRNA. **(C)** and **(D)** Inhibition of invasion of SW480 and LoVo cells by SNHG1 siRNA. Data are shown as mean ± SD. The experiments were all repeated at least three times. ^**^ *P*< 0.01 compared with Si-NC.

To further examine the effect of SNHG1 in cell migration and invasion, transwell assays were conducted in SW480 cells and LoVo cells. Downregulation of SNHG1 dramatically diminished SW480 and LoVo cell migration capability compared with the Si-NC group (^**^P<0.01, Figure 4A and 4B). We further performed transwell invasion assay to explore the effect of SNHG1 on the invasiveness of colorectal cancer cells. These results confirmed that downregulation of SNHG1 robustly inhibited the ability of cell invasion compared with the Si-NC group (^**^P<0.01, Figure 4C and 4D).

### Silencing of SNHG1 impaired Wnt/β-catenin signaling activation in colorectal cancer

**Figure 5:**
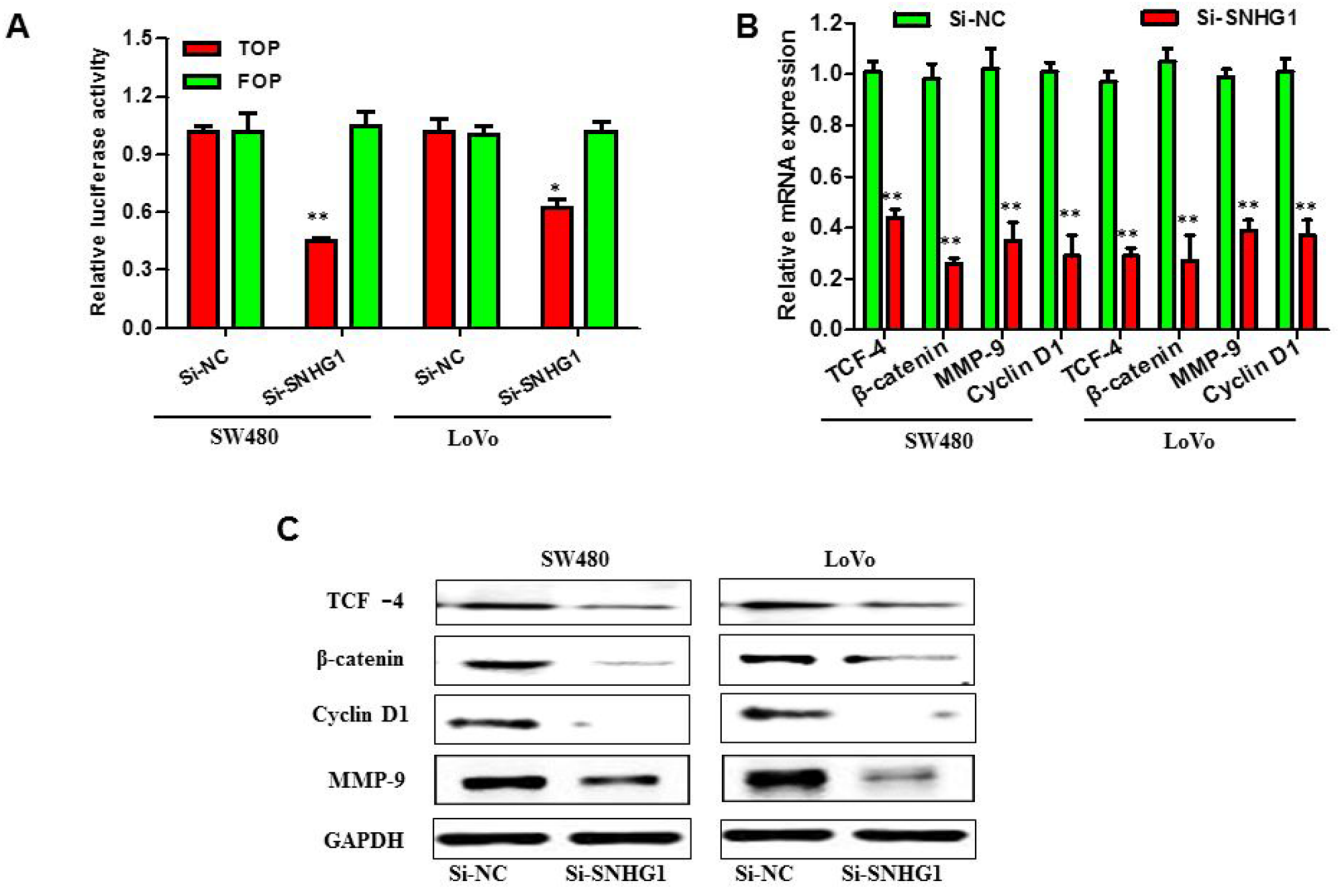
SNHG1 facilitated colorectal cancer malignant progression through by Wnt/β-catenin signaling pathway SW480 and LoVo cells were transfected with Si-SNHG1 and Si-NC for 48 h. **(A)** Luciferase reporter assay using TOP flash vectors was carried out to detect β-catenin transcription factor/lymphoid enhancer binding factor (TCF/LEF) promoter activity; FOP flash has mutated TCF binding sites, acting as a negative control. SNHG1 siRNA treatment inhibited β-catenin TCF/LEF promoter activity. **(B)** and **(C)** qRT-PCR and Western blot analysis of proteins (β-catenin,TCF-4) in the WNT/β-catenin signaling pathway and downstream targets of WNT/β-catenin signaling pathway, such as cyclin D1 and MMP-9. Each assay was performed in triplicate. Data are mean ± SD. ^*^P<0.05, ^**^P<0.01.

Recent studies have reported that SNHG1 may stimulate the progression of cancer through modulating the WNT signaling pathway in the NSCLC [17] and WNT signaling pathway is also critical in the initiation, progression and metastasis of colorectal cancer [21]. Therefore, we speculated that SNHG1 might serve a principal role in regulating the activation level of the WNT/β-catenin signaling pathway in the colorectal cancer. In order to examine the association between SNHG1 expression level and the activation level of the WNT/β-catenin signaling pathway, TOP/FOP flash reporters (Howard Hughes Medical Institute, University of Washington, USA) were performed to evaluate the effects of SNHG1 on the WNT/β-catenin signaling pathway in SW480 and LoVo cells. The change of cell luciferase activity was shown in Figure 5A. While SNHG1 expression was reduced, the activation of the WNT/β-catenin signaling pathway was significantly inhibited. We then detected the expression levels of TCF-4 using qRT-PCR and Western blot analysis. As is known, the WNT/β-catenin signaling pathway plays an essential role in the regulation of cell proliferation. Thus, the expression of β-catenin and a few of the downstream genes of the WNT/β-catenin signaling pathway, for instance, CCND1 and MMP9 were detected by qRT-PCR and Western blot assay. By data, we found that the expression of TCF -4, β-catenin, cyclin D1 and MMP-9 was robustly down-regulation when SNHG1 was silenced in SW480 and LoVo cells (Figure 5B and 5C). All results above demonstrated a novel correlation between SNHG1 expression and the activation level of the WNT/β-catenin signaling pathway in the colorectal cancer.

## DISCUSSION

In recent years, the examination of cancer-related lncRNAs and analysis of their clinical significance and biological functions in cancers have raised immense research interests from researchers around the world [10, 22]. The dysregulation of lncRNAs have also been exhibited to result in the onset and development of many types of cancers, including colorectal cancer. For instance, long noncoding RNA BLACAT1 indicates a poor prognosis of colorectal cancer and affects cell proliferation by epigenetically silencing of p15 [23]. Moreover, up-regulated expression of SPRY4-IT1 predicts poor prognosis and promotes malignant development of colorectal cancer by targeting epithelial-mesenchymal transition [24, 25]. Additionally, lncRNA H19 identified as an independent prognostic factor of CRC patient survival, regulates essential Rb-E2F and CDK8-β-catenin signaling in colorectal cancer [26]. Other important lncRNAs, such as HOTAIR and CCAT2 have been showed that they have played critical roles in the development of colorectal cancers and are associated with poor prognosis in CRC [27–29]. These above reports indicated that lncRNAs could serve as diagnostic and prognostic biomarkers in colorectal cancer. However, the biological function and clinical significance of lncRNAs in colorectal cancer cancer are not well understood.

SNHG1 has been shown to be strikingly increased in several cancer and up-regulation of SNHG1 indicated the poor prognosis of tumor patients, and silencing of SNHG1 expression contributed to suppression of cancer cell proliferation or growth, diminishment of migration and invasion, inducement of apoptosis in non-small cell lung cancer, prostate cancer, glioma, primary esophageal cancer cells and hepatocellular carcinoma [13, 14, 16, 17, 30]. However, to our knowledge, the clinical significance and biological function of lncRNA SNHG1 in colorectal cancer remains largely unknown. Our data in the present study showed remarkable increase in SNHG1 expression in colorectal cancer tissues compared with adjacent non-cancerous colorectal tissues. Further analysis showed that the up-regulation of SNHG1 was positively associated with the tumor diameter, histological differentiation, TNM stage, lymph node metastasis, depth of invasion.

In addition, Kaplan-Meier and Cox regression analysis was used to explore the prognostic value of SNHG1. Our data revealed that the patients with high SNHG1 expression had poorer overall survival and progression-free survival than those with the low SNHG1 expression. The multivariate analyses suggested that the high SNHG1 expression could be served as a potential independent prognostic factor for overall survival and progression-free survival of colorectal cancer patients. These data elucidated that high SNHG1 expression could be used as a novel predictive biomarker of poor prognosis in colorectal cancer and might be a potential therapeutic target for diagnosis and therapy of CRC.

Recent reports suggest that lncRNAs play crucial role in cancer growth and metastasis. Long non-coding RNA GAS5 inhibits cell proliferation, induces G0/G1 arrest and apoptosis, and functions as a prognostic marker in colorectal cancer [31]. Over-expressed long noncoding RNA HOXA11-AS promotes cell cycle progression and metastasis in gastric cancer [32]. We then examined the function of SNHG1 in CRC cells by loss of function methods. Inhibition of SNHG1 dramatically restrained the growth of colorectal cancer cells by MTS assay and colony formation, arrested cell cycle and induced the cell apoptosis by flow cytometry, as well as attenuated migration and invasion of CRC cells by transwell assay *in vitro*, indicating that SNHG1 may serve as an oncogene that promotes CRC malignant progression.

Moreover, mounting evidence indicates that lncRNA may be involved in CRC development and progression through the activation of the WNT/β-catenin signaling pathway. Han et al. reported that lncRNA CRNDE could regulate the progression and chemoresistance of CRC via modulating the activity of Wnt/β-catenin signaling [33]. Similarly, Ma et al. showed that long non-coding RNA CCAL regulates colorectal cancer progression by activating Wnt/β-catenin signalling pathway via suppression of activator protein 2α [34]. Overexpression of long non-coding RNA-CTD903 inhibits colorectal cancer invasion and migration by repressing Wnt/β-catenin signaling and predicts favorable prognosis [35]. Additionally, long non-coding RNA CASC11 interacts with hnRNP-K and activates the WNT/β-catenin pathway to promote growth and metastasis in colorectal cancer [36]. Therefore, we further explored whether SNHG1 facilitated the growth and metastasis of CRC by activating the WNT/β-catenin pathway. We performed a representative luciferase reporter assay, TOP/FOP Flash, to probe the activity of TCF- dependent transcription. As a result, SNHG1 significantly stimulated the activation level of the WNT/β-catenin signaling pathway. Because β-catenin was not only acting as a part of the cadherin-based adhesion protein complex, but also as an important endocellular effector for the WNT/β-catenin signaling pathway. So, that is why we think that the effects of SNHG1 on tumor cell proliferation and metastasis might be associated with β-catenin regulation. Our results further demonstrated that silencing of SNHG1 inhibited β-catenin expression and suppressed the activation level of the WNT/β-catenin signaling pathway. Besides, in terms of mRNA and protein expression level, our data displayed that SNHG1 could also regulate several downstream target genes of the WNT signaling pathway, for instance, MMP9, TCF4, and CCND1. Based on our results, we therefore concluded that SNHG1 could be involved in the WNT/β-catenin signaling pathway. However, other additional mechanisms might exist in the regulation of β-catenin expression to a large extent, such as modulation by miRNA sponges to affect the expression of β-catenin, or with the co-regulation of other signaling pathways. Hence, our results identified a new clue for comprehending the molecular mechanism of CRC development and made a way for the diagnosis and treatment of CRC.

Altogether, we provided the evidence for the first time that the SNHG1 expression is predominantly augmented in colorectal cancer compared to paired-adjacent non-tumorous tissues, and demonstrated that SNHG1 may play a key oncogenic role as a predictor of poor survival and a negative prognostic factor for patients with colorectal cancer. We also elucidated that knockdown of SNHG1 may decrease proliferation, arrest cell cycle, promote apoptosis, limit migration and invasion of colorectal cancer cells. Notably, mechanistic analysis revealed a novel SNHG1-β-catenin-WNT signaling pathway regulatory network in CRC. These new findings suggested that SNHG1 may be used as a potential prognostic and therapeutic target of colorectal cancer.

## MATERIALS AND METHODS

### Human tissue samples collection

108 pairs of colorectal cancer tissues and the pair-matched adjacent non-tumor tissues were acquired from the Zhejiang Cancer Hospital between Jun 2011 and Feb 2012. All the patients received partial or radical cystectomy. All specimens were frozen immediately in liquid nitrogen and stored at −80°C until RNA extraction. None of the patients from whom the samples were obtained had undergone preoperative local or systemic treatment. Informed consents were collected from all patients and this study was approved by the Clinical Research Ethics Committee at the Zhejiang Cancer Hospital.

### Cell lines and cell culture

Normal intestinal mucous cell line (CCC-HIE-2) were purchased from ATCC (American Type Culture Collection, Manassas, VA, USA). Human CRC cell lines SW480, HCT116, HT-29, LOVO, CaCO-2 cells were obtained from the Chinese Academy of Sciences (Shanghai, China). All cells were maintained in Dulbecco’s Modifid Eagle Medium (DMEM; Gibco, Grand Island, NY, USA) supplemented with 10% fetal bovine serum (FBS; Gibco) and 1% antibiotics (100 units/mL penicillin and 100 μg/ mL streptomycin sulfates) at 37°C with an atmosphere of 5% CO_2_ in incubator. All cell lines have been tested and authenticated by DNA (short tandem repeat genotyping) profiling before use.

### RNA preparation, quantitative real time RT-PCR (qRT-PCR) analysis

The total RNA was extracted from collected tissues or cultured cells with Trizol reagents (Invitrogen, Shanghai, China) according to the manufacture’s instructions. The total RNA was reverse transcribed into cDNA using a Reverse Transcription Kit (Takara, Dalian, China) according to the manufacture’s guide. qRT-PCR was performed on SYBR^®^ Green One-Step Real-Time RT-PCR Master Mixes(Thermo Fisher, Shanghai, China) on Agilent Stratagene Mx3000P Quantitative PCR System (Agilent Technologies, USA) according to the guide. Results were normalized to the expression of glyceraldehyde-3-phosphate dehydrogenase (GAPDH). The PCR primers were as follows: SNHG1 primers forward: 5′-AGGCTGAAGTTACAGGTC-3′, reverse:5′-TTGGCTCCCAGTGTCTTA-3′; Cyclin D1 primers forward:5′-GAGACCATCCCCCTGACGGC-3′, reverse: 5′-TCTTCCTCCTCCTCGGCGGC-3′; β-catenin primers forward: 5′-TGCAGTTCGCCTTCACTATG-3′, reverse: 5′-ACTAGTCGTGGAATGGCACC-3′; TCF-4 primers forward: 5′-ATGGCAAATAGAGGAAGCGG-3′, reverse: 5′-TGGAGAATAGATCGAAGCAAG-3′; GAPDH primers forward: 5′-TGCACCACCAACTGCTTAGC-3′, reverse: 5′-GGCATGGACTGTGGTCATGAG-3′. All primers were purchased from GenScript Co. Ltd (Nanjing, China). All qRT-PCR reactions were done in triplicate. Relative quantification of tested gene expression was calculated using the comparative cycle threshold (CT) (2^−ΔΔCt^) method.

### Cell transfection

The small interfering RNAs (siRNAs) specifically targeting SNHG1 and scrambled negative control were synthesized from GenePharma Co. Ltd (Suzhou, China). The siRNA sequences for SNHG1 were si-SNHG1-1, 5′-CAGCAGTTGAGGGTTTGCTGTGTAT-3′, si-SNHG1-2, 5′-TTCAACAGCTAGGTTGTCCTT-3′. si-RNAs were transfected into colorectal cancer cells cultured in six-well plates using Lipofectamine 2000 transfection reagent (Invitrogen, Carlsbad, CA, USA) at a final concentration of 50 nM according to the provided instructions.

### Cell proliferation assay

Cell proliferation detection kit (MTS) (cellTiter96AQ, Promega, Madison, WI) was used to evaluate the cell viability according to the manufacturer’s protocol. Cells were seeded into 96-well plates 24 h after transfected specific siRNAs at a density of 3×10^3^ cells/well. Cell viability was detected at different time points (24, 48, 72, and 96 h). Absorbance was measured at 490 nm with SpectraMax^®^ M5 (Thermo Fisher Scientific). Experiments were performed in triplicate.

### Colony formation assay

Transfected cells were plated in 6-well plates at a density of 500-800 cells per well and incubated for two weeks. At the end of incubation, the cells were gently washed with PBS and then fixed with methanol for 10 min and stained with 0.1% crystal violet (Sigma). The number of colonies (more than 50 cells as a colony) was captured with a microscope and counted by Image J software (NIH, Bethesda, MD) from three independent replicates.

### Cell apoptosis and cycle flow cytometry assay

The cell apoptosis was determined by the Annexin V-FITC apoptosis kit (BD Biosciences, USA) according to the manufacturer’s guide. Briefly, cells were harvested and washed with PBS, stained in 500μl binding buffer with 5μl of Annexin V-FITC (BD Biosciences) for 15 min and 5μl PI for another 5 min. Apoptosis were measured by flow cytometry (BD Biosciences, FACS Calibur). For the cell cycle analysis, cells were harvested and washed with cold PBS, fixed with 75 % ice-cold ethanol at −20°C overnight, stained with propidium iodide (PI) dye solution (Sigma) supplemented 0.1 mg/ml Rnase A (Promega) for 30 min at room temperature. The cell cycle distribution was analyzed by flow cytometry (FACSCalibur, BD Biosciences).

### Histone DNA ELISA assay

The cell apoptosis was assessed by the Cell Apoptosis Histone-DNA ELISA Kit (Roche Applied Science, Shanghai, China) according to the manufacturer’s instructions. Detailed procedures can be found in other studies [37]. Briefly, the cells were lysed and the cell lysates were collected and incubated in well coated with anti-Histone antibody. Samples were then incubated with horseradish peroxidase–conjugated anti-DNA antibodies followed by color reaction with ABTS substrate. The absorbance of the samples were measured with a microplate reader at 405 nm.

### Migration and invasion assay

This assay was performed as described previously [19]. Migration and invasion experiments *in vitro* were performed in the 24-well plate of 8-μm transwell chamber (BD Biosciences, USA) with or without Matrigel (1:3 mixed with PBS; BD Falcon^™^). Cells transfected (1 × 10^5^ cells per well) were suspended in 100μl serum-free medium and then plated onto the top chamber in the 24-well plate, and the lower chamber of each well insert was filled with 600μl serum-containing medium. After 24h of incubation at 37°C, the cells that migrated or invaded into the lower chambers were fixed with 4% paraformaldehyde, washed with PBS, stained with crystal violet and then counted under a light microscope (Olympus, Tokyo, Japan) at ×100 magnification in five randomly selected fields across the center and the periphery of the membrane. Experiments were performed in triplicate.

### Western blot analysis

Cells were lysed in RIPA lysis buffer with protease and phosphatase inhibitors (1 mM Na3VO4, 10 mM NaF, 1 mM phenylmethanesulfonyl fluoride, 2 μg/ml aprotinin). Protein concentrations were measured using the Bio-Rad protein assay (Bio-Rad Laboratories). Equal amounts of the protein were denatured in sample buffer and then electrophoresed by 5-10% SDS-PAGE, transferred to the nitrocellulose membrane (iBlot^®^ 2 Transfer Stacks, Thermo Fisher Scientific, USA) under 100 V for 2 h. Membranes were blocked for 1 hour in TBST buffer containing 5% BSA and then incubated with the following primary antibodies overnight at 4°C: anti-GAPDH antibody (Rabbit mAb #5174, Cell Signaling Technology); anti-TCF-4 antibody (Rabbit mAb # PA1-10041, Thermo Fisher Scientific), anti-β-catenin (ab6302, Abcam); anti-cyclin D1 antibody (ab16663, Abcam); anti-MMP-9 antibody (Rabbit mAb #3969, Biovision). After washed in TBST, the membranes were further incubated with a secondary antibodies that were 10,000-fold diluted. Enhanced chemiluminescence (Thermo Fisher Scientific) solution was added onto the membranes and signals were detected with an Odyssey Infrared Imaging System (LI-COR).

### Statistical analysis

All data were expressed as the means ± standard deviation (SD). The Fisher exact test or Student’s t test was used for differences comparisons between two independent groups, while difference among multiple groups was analyzed using one–way ANOVA followed by Bonferroni’s multiple comparisons test. Kaplan-Meier survival cures were generated for colorectal cancer patients with lower or higher SNHG1 expression, and the difference was analyzed by log-rank test. Univariate and multivariate Cox proportional hazards model was used to evaluate the survival data. Data was analyzed using GraphPad Prism 6.0 (GraphPad Software, San Diego, CA, USA). Statistically significant differences were defined as ^*^P <0.05, ^**^P<0.01 and ^***^P<0.001.
